# Is cervical swab an efficient method for developing a new noninvasive prenatal diagnostic test for numerical and structural chromosome anomalies?

**DOI:** 10.3906/sag-2009-347

**Published:** 2021-06-28

**Authors:** Erkan YURTCU, Deniz KARÇAALTINCABA, Hasan Hüseyin KAZAN, Halis ÖZDEMİR, Meral YİRMİBEŞ KARAOĞUZ, Pınar ÇALIŞ, Gülsüm KAYHAN, Sezen GÜNTEKİN ERGÜN, Ferda PERÇİN, Merih BAYRAM, Mustafa Necmi İLHAN, Gamze BİLGİLİ, Tuğrul KAYMAK, Mehmet Ali ERGÜN

**Affiliations:** 1 Department of Medical Biology, Faculty of Medicine, Başkent University,Ankara Turkey; 2 Department of Obstetrics and Gynecology, Faculty of Medicine, Gazi University, Ankara Turkey; 3 Department of Medical Genetics, Faculty of Medicine, Gazi University, Ankara Turkey; 4 Department of Obstetrics and Gynaecology, Sami Ulus Women’s and Children’s Health Training and Research Hospital, Ankara Turkey; 5 Department of Medical Biology, Faculty of Medicine, Hacettepe University, Ankara Turkey; 6 Department of Public Health, Faculty of Medicine, Gazi University, Ankara Turkey; 7 Mycotoxin Analysis Laboratory, Ministry of Agriculture and Forestry, Ankara Turkey

**Keywords:** Cervical swab, CMA (comprehensive meta-analysis), fluorescence-activated cell sorting (FACS), magnetic-activated cell sorting (MACS)

## Abstract

**Background/aim:**

Prenatal diagnosis is vital to obtain healthy generation for risky pregnancies. There have been several approaches, some of which are routinely applied in clinics to evaluate the possible prenatal deficiencies and/or diseases. In the present study, we aimed to isolate the fetal cells from endocervical samples and try to identify possible anomalies which were proved by Amniocentesis (AS) and chorionic villus sampling (CVS) methods.

**Materials and methods:**

Endoservical specimens were collected from 100 pregnant women. Cells were separated in parallel by fluorescence-activated cell sorting (FACS) and magnetic-activated cell sorting (MACS) using human leukocyte antigen (HLA) G233 and placental alkaline phosphatase (PLAP) antibodies. CMA (comprehensive meta-analysis) were carried out and male fetuses were confirmed with Sex determining region Y (SRY) amplification.

**Results:**

The percent of HLA G233 and placental and placental alkaline phosphatase (PLAP) positive cells were 4.55% and 84.59%, respectively. The percent of cells positive for both markers was 14.75%. CMA analyses were not informative. (SRY) was amplified in 67% of the samples.

**Conclusion:**

However, the success rate of the both cell sorting and scanning of DNA anomalies by aCGH and/or RT-PCR was limited, preventing the applicability of this proposal in the clinics. Still, the success of the proposed method depends on the development of the novel fetal cell-specific antibodies and the improvements in the sorting systems.

## 1. Introduction

Prenatal diagnosis can be defined as the earliest detection of genetic diseases that may occur in the fetus during pregnancy. The target population in prenatal diagnosis can be classified as advanced maternal age (≥35 years), chromosomal anomaly in previous pregnancies or spouse, presence of genetic disease(s) in the family, congenital anomalies, mental retardation, and increased risk in diagnostic tests. Prenatal diagnosis methods are divided into two groups as invasive and noninvasive methods. CVS, amniocentesis, and cordocentesis are included in the invasive methods, whereas ultrasonography (USG), biochemical screening tests, and free fetal DNA in maternal blood are the noninvasive methods. The fetal loss rates associated with AS and CVS have been reported as 0.1%–0.9% and 0.2%–1.3%, respectively. Regarding other interventional method, cordocentesis, the risk of fetal loss is high (1.3%) [1,2].

Although the noninvasive diagnostic methods, such as biochemical screening tests and USG have no risk of fetal loss; the detection rate varies between 50%–95% with false positive rate of 5% [3].

American College of Medical Genetics Genomics (ACMG) has proposed the use of extracellular free fetal DNA for noninvasive prenatal screening tests (NIPS) since 2013 [4]. Approximately 10% of DNA in maternal serum is of fetal origin [3]. Fetal DNA ratio is of great importance in terms of diagnosis. Comparing chromosome microarray studies and noninvasive program stimulation (NIPS), the success rate was reported to be low, while the fetal DNA rate was below 5% and high above 27% [5]. However, unbalanced translocations, deletions, and duplications cannot be detected with fetal DNA obtained from maternal blood. Also, single gene mutation analysis cannot be performed [3]. In addition, NIPS does not show neural tube defects (NTD). Maternal alpha fetoprotein analysis should be performed for diagnosis. NIPS cannot replace USG results in terms of nuchal thickness, twin pregnancy, placental anomalies, and congenital anomalies [6]. Finally, it does not give any information about late pregnancy complications [5].

For the analysis of the fetal genome, obtaining fetal cells in a noninterventional way is crucial. Since 1970s, fetal cells have been shown to be available in endocervical canal by uterine aspiration, endometrial biopsy, and lavage. In pregnant women, cervical mucus contains trophoblasts, and these cells are detectable in the endocervical canals of pregnant women at 7-13th of gestational weeks [7]. 

Although the detection rate of fetal cells in endocervical samples varies, it can reach 70%–98%. Chromosome and single gene diseases can be determined in fetal transcervical cells using fluorescence in situ hybridization (FISH), polymerase chain reaction (PCR), and quantitative fluorescence-polymerase chain reaction (QF-PCR) methods [8,9]. For all these reasons, obtaining transcervical fetal cells has been proposed as a noninvasive prenatal diagnosis method. However, depending on the gestational age, the method of sampling, the skills of the operator, and whether the pregnancy is normal or abnormal, fetal cells can be obtained by transcervical methods at the rate of 40%–90% [9]. 

The aim of this study is to determine the structural and numerical anomalies pertaining to 21, 13, 18, X, and Y chromosomes, by obtaining fetal cells from pregnant women in early gestation weeks by endocervical lavage with chromosomal microarray (CMA) technique.

## 2. Materials and methods

The study was approved by the ethical committee of Keçiören Eğitim ve Araştırma Hastanesi (2012-KAEK-15/1498).

### 2.1. Study population and design

The study was carried out during the period from 26.11.2018 to 22.8.2019, and a total of 100 pregnant women were included in the study. 

### 2.2. Endoservical sampling

Cervical swabs were taken by cytobrush from pregnant women between 12–18th gestational week who admitted for invasive genetic testing in the Gazi University Department of Obstetrics and Gynecology. Only swabs of pregnant women known to have a male fetus were studied. Fetal cells from swabs from pregnant women were sorted by both fluorescence activated cell sorter (FACS) and magnetic-activated cell sorting (MACS).

### 2.3. Cell sorting

FACS: The cells from the cervical swab were separated according to the cell surface marker expression via the FACS. Briefly, after the cells obtained by cervical swab have been taken into PBS, samples have been incubated with FITC-labeled HLA-G G233 clone specific monoclonal antibody (BD, Germany) at room temperature for 30 min. PerCp-labeled PLAP antibodies were used for double staining. Cells were stained with PLAP antibodies (BD, Germany) for additional 30 min after HLA-G 233 labelling. After washing with PBS, the cells were sorted via flow cytometry by applying voltage specific to the monoclonal antibody label in the appropriate fluorophore excitation in the FACS device. Numerical analysis of the samples was carried out by simultaneously recording parameters such as the number of cells showing the HLA-G 233 positivity and the total number of cells through the software of the FACS device.

MACS: Cell staining for MACS were made similar to staining for FACS. The antibodies used MACS were labeled with secondary antibodies attached to magnetic beads instead of fluorochromes. Sorting process was carried out in magnetic environment using according to the supplier’s (Milteny, Germany) instructions.

### 2.5. DNA isolation

Commercial DNA isolation kit (QIAamp DNA Micro Kit, Qiagen, Germany) was used to obtain DNA from fetal cells separated by both FACS and MACS. In cases where the amount of DNA was not sufficient, the whole genome amplification was performed with the PicoPLEX WGA kit (Rubicon Genomics, USA) kit.

### 2.6. CMA analysis

CMA analysis has been conducted for 25 patients who had been admitted in the 1st trimester in the Department of Obstetrics and Gynecology, Gazi University, Ankara, Turkey. In these patients, the results from isolated cells were confirmed by that of amniocentesis material. 

With restriction enzymes, DNAs to be obtained from fetal cells were cut between 2–16 h. Samples were prepared on the ice with appropriate amounts of buffer, ligase enzyme, and adapter suitable for the restriction enzyme, and a mixture of DNA was mixed with DNA for 3 h in a suitable incubation program. PCR was performed by diluting the products after ligation. For purification, the appropriate amount of magnetics bead solution was added to the PCR reaction; after the mixture was kept on the magnetic stand and the DNA collected at the bottom of the tube was taken with the appropriate micropipette, the concentrations of the products to be obtained at the end of the process were measured in the spectrophotometer. After the purified PCR product was diluted with the fragmentation solution, a 45 min fragmentation reaction was performed in the thermal cycler. Then, marking mixture was prepared with materials, such as enzyme and buffer on ice and added to the fragmented DNA mixture, and a marking reaction was carried out in the thermal cycler for 4 h. After adding hybridization solutions to the marked DNA sample and incubating, the arrays were injected with a micropipette. Arrays were placed in the hybridization oven, and hybridization was performed at 49 °C with a rotation of 60 rpm for 16–18 h. After hybridization, the liquid in the array was withdrawn with a micropipette, and solutions with dyes were injected into the array washing station. After the washing process, scanning was carried out with the help of the software. Then the data obtained were analyzed with Agilent CytoGenomics software.

### 2.7. SRY analysis with Real time PCR

The confirmation of male fetuses had been performed with an in-house real-time PCR method. We designed SYBR-Green based SRY amplification protocol with the primers below:

Forward primer 5’-GAGAATCCCAGAATGCGAAA-3’

Reverse primer 5‘-GTAAGTGGCCTAGCTGGTGCT-3’

## Results

### 3.1. FACS-MACS

**Figure 1:**
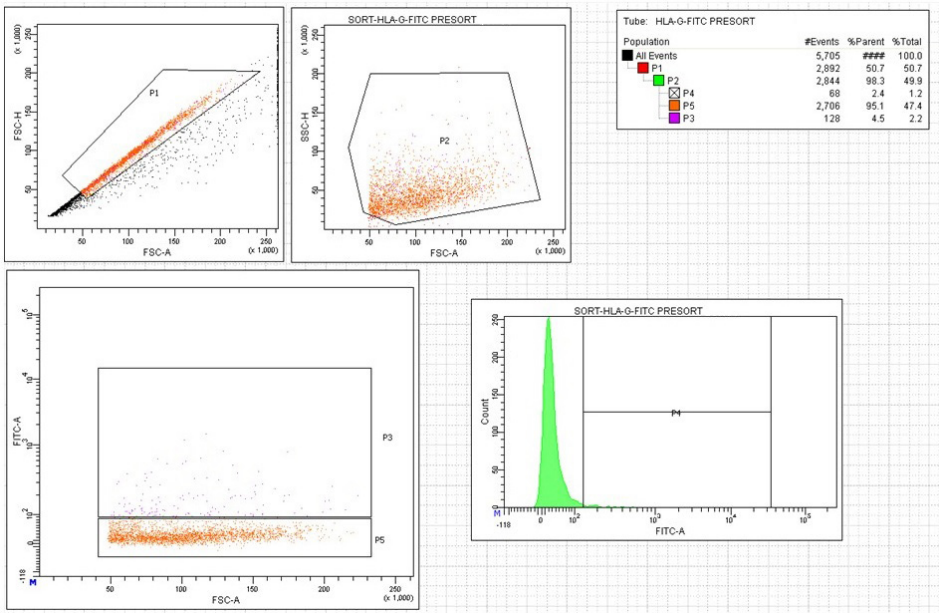
Gates of FACS for HLAG233.

The swab materials taken from the pregnant women were divided into two groups. The parallel sorting was done with FACS and MACS. The representative figure of gate strategy was given in Figure 1, results of the FACS were given in Table. 

**Table:** The cell counts of patients who underwent FACS.

	Total	HLAG+ (%)	PLAP+ (%)	HLAG+ and PLAP+(%)
Mean	2529390	123062 (4.55)	489833 (84.59)	124908 (14.75)
Min.	7500	900	0	0
Max.	12890000	1880000	1800000	2044000
Median	1000000	40500	134500	15750

### 3.2. CMA analysis

**Figure 2:**
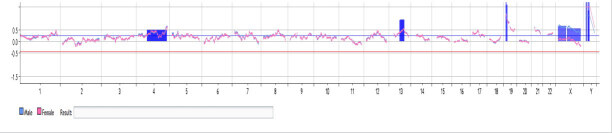
The CMA results indicating trisomy of chromosomes 4, 13, 19, and the sex chromosomes.

The CMA analysis in 25 patients did not reveal compatible results with the amniocentesis regarding structural and numerical anomalies. Only in one patient, we found compatible results regarding Y chromosome, but additional chromosomal abnormalities were also detected that were not compatible with the amniocentesis results (Figure 2).

### 3.3. Amplification of SRY with real time PCR 

**Figure 3:**
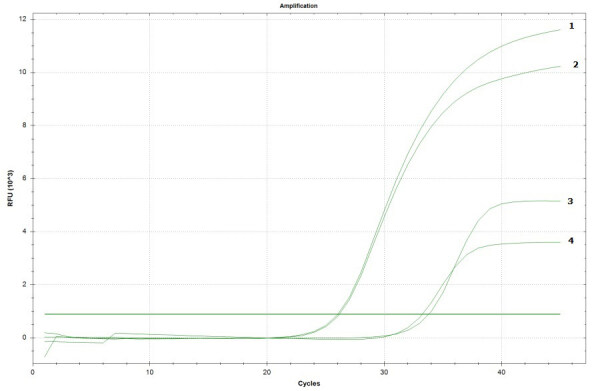
Representative figure for SRY amplification for male control (1), DNA from AS material (2), and DNA from sorted cells (3–4).

The success rate of SRY amplification from cells obtained from both sorting methods was 67%. Amplification plots of representative samples are shown in Figure 3. The corresponding figure underlies the amplification curves for SRY region in the DNAs obtained from sorted cells with cytotrophoblast more than 30 in the presence of male control and isolated DNA from AS material with cytotrophoblast more than 20. Although this result cannot totally clarify the success rate of the approach, it may imply that cell sorting could be successful in terms of isolation of fetal cells without known separation ratio.

## 4. Discussion

More than 100 years ago, Schmorl showed that trophoblastic cells can be found in the uterine vein. About 50 years ago, it was suggested that these cells can also be found in the uterine cavity and cervical canal [10]. Some researchers have attempted to isolate fetal cells from maternal blood. However, the ratio of fetal cells in maternal blood is only 1–2 cells in 1 mL of blood [11]. Others made efforts to collect fetal cells from the uterine canal. For this purpose, morphological identification and micromanipulation were used to isolate trophoblasts from endocervical samples. After that, fetal cells were separated using specific monoclonal antibodies [12]. HLA-G protein is first expressed in trophoblast cells of the anchoring villi that have differentiated into invasive extravillous trophoblasts HLA-G, which is expressed by human extravillous trophoblast cells and is not expressed in adult tissues of the cervix or uterine cavity. HLA-G has proven to be a reliable protein marker to identify trophoblast cells collected from the cervix [7]. The frequency of HLA-G positive cells in the normal IUP was 1 in 2000 cells [9]. In a previous study, Bulmer et al. collected transcervical lavage samples from pregnant women and identified fetal cells by immunostaining using McAb against anti-HLA-G (G233) [8]. They showed that about 50% of the samples contained cytotrophoblastic cellular elements with a variable number. They found that the specificity of this antibody is high, but its sensitivity is low; thus, they suggested that it would be better to use a monoclonal antibody panel [8]. In this study, we determined HLAG positivity in only 4.55% for our samples. 

By analyzing the term placental villi, placental and PLAP expression has been shown in both syncytiotrophoblasts and cytotrophoblast. In a previous study, Miller et al. [13] recovered cells from pregnant women by transcervical flushing and aspirating. Syncytiotrophoblasts were morphologically identified in 29% of pregnancies. The authors identified fetal cells with monoclonal antibodies, including PLAP, in only 50% of cases. These cells were small, round and has hyperchromatic nucleus and morphologically different from syncytiotrophoblasts. The authors included pregnant women with male fetus in the study for easy follow-up of fetal cells. However, they were able to show the presence of Y chromosome in only 62% and 60% of the cases by PCR and In-situ hybridization, respectively [13]. In this study, PLAP positivity of fetal cells was 84.59%. 

We used two antibodies together to separate higher purity fetal cells. The combined positivity for both markers was found to be 14.75%. This means we were able to separate HLA-G/PLAP positive cells in the endocervical samples. However, both the nonspecificity of these surface markers, and the diversity of the number of the isolated cells lowered the success rate of our results. Also, as mentioned above, morphological differences of staining cells may affect sorting efficiency of FACS.

Other factors that affect the success rate are the capabilities of the operator and the method used for collection. In the current study, two obstetricians performed sampling procedures and cells were collected with endocervical brush. The presence of fetal cells shed from the placenta in the cervical mucus is another factor negatively affecting the isolation. So, we propose that these markers used in FACS/MACS were not 100% efficient in fetal cell isolation.

Using an artificial mosaicism series, we found that oligonucleotide aCGH using specific analysis parameters could accurately measure levels of mosaicism down to 10% and that the degree of mosaicism could be predicted from fluorescence ratios [14]. Thus, aCGH, which is based on genomic DNA extracted directly from uncultured peripheral blood, may be more likely to detect low-level mosaicism for unbalanced chromosome abnormalities than traditional cytogenetic techniques [15]. The false negativity in the aCGH can also be attributed to the mosaicism of the fetal cells. The techniques regarding whole genome amplification also did not change the results due to the low level of mosaicism in the cervical lavage sample. Our incompatible results between CMA and amniocentesis may be attributed to the maternal cell contamination or low frequency of the fetal cells.

Finally, we included SRY analysis in male fetuses. The results were promising with respect to the CMA analysis. We also think the nonspecificity of the markers resulted in false negative results in real time PCR analysis.

In conclusion, due to the failure of isolation of fetal cells from endocervical lavage by FACS, we think endocercival swab method is not an efficient method for detecting numerical and structural chromosome anomalies with aCGH. So, we think regarding noninvasive techniques, maternal blood will be preferred more with respect to endocervical lavage. Still, the success of the applied approach could be maximized by novel antibodies specific to the fetus and improvements in the cell sorting systems. 

## Informed consent

The study was approved by the ethical committee of Keçiören Training and Research Hospital (2012-KAEK-15/1498).
